# The Efficacy of Sample Storage Envelopes in Collecting Scales From the Skin and Nails for Mycology Laboratory Diagnosis of Superficial Fungal Infection

**DOI:** 10.1155/2024/6325772

**Published:** 2024-11-07

**Authors:** Lalita Matthapan, Sumanas Bunyaratavej, Charussri Leeyaphan, Pantaree Kobkurkul, Thrit Hutachoke, Supisara Wongdama, Suthasanee Prasertsook, Bawonpak Pongkittilar, Chatisa Panyawong, Waranyoo Prasong, Akkarapong Plengpanich

**Affiliations:** Department of Dermatology, Faculty of Medicine Siriraj Hospital, Mahidol University, 2 Wanglang Road, Bangkok Noi, Bangkok 10700, Thailand

**Keywords:** dermatophytosis, onychomycosis, sample storage envelopes, superficial fungal infections

## Abstract

**Background:** Fungal cultures are unavailable in many hospitals. The development of an effective sample storage solution for timely transportation would improve management of patients with superficial fungal skin and nail infections.

**Objectives:** This study aimed to evaluate the efficacy of sample storage envelopes to preserve skin and nail samples for timely microscopic examination and culture for superficial fungal infections.

**Methods:** Patients aged 18 years and above with suspected superficial fungal infections were enrolled. The samples were divided into four envelopes. The baseline 20% potassium hydroxide (KOH) examination and fungal culture served as reference points. The stored samples were reexamined on Days 3, 7, 14, and 28.

**Results:** The study included 90 patients with suspected superficial fungal infections (45 skin and 45 nail lesions). Reference KOH examinations showed branching septate hyphae in 36 (80.0%) for skin and 35 (77.8%) for nail infections. Reference fungal cultures were positive for the growth of dermatophyte and nondermatophyte molds in 34 (75.6%) and 28 (62.2%) in skin and nail infections, respectively. Sample storage envelopes maintained 100% sensitivity and specificity for up to 28 days with KOH examination for both skin and nail samples. On Day 28, the fungal culture sensitivity was 70.6% for the skin and 64.3% for the nail samples, with specificities of 100.0% and 88.2%, respectively.

**Conclusions:** Sample storage envelopes effectively maintained diagnostic accuracy for up to 28 days with KOH examination for both skin and nail samples. Given the high specificity even on Day 28 for fungal culture, transferring samples within 28 days remains a reliable practice.

## 1. Introduction

Superficial fungal infections can be caused by various groups of fungi. The most common pathogens involved are dermatophytes, *Candida* spp., and *Malassezia* spp. However, infections by nondermatophytes, including *Neoscytalidium dimidiatum* and *Fusarium* spp., are also possible, particularly in endemic areas [1–3]. These infections mainly affect the skin, hair, and nails. Superficial fungal infections are considered a common global disease, with an increasing prevalence of approximately 20%–25% [4].

Differentiating superficial fungal infections from other skin diseases solely on the basis of skin manifestations can be challenging. Therefore, it is crucial to have accompanying laboratory results. These include conventional methods such as direct microscopy and fungal culture, as well as advanced DNA-based techniques such as PCR. The selection of appropriate tests depends on several factors, including local resources, costs, and availability [5].

Potassium hydroxide (KOH) examination and fungal cultures remain standard diagnostic methods for superficial fungal infections. However, these methods require experienced microscopists or technicians to interpret the results with high sensitivity and minimize false negative results. Furthermore, qualified laboratories that are capable of performing fungal culture and identification are limited to only tertiary hospitals [6]. To address these challenges, the collection of samples and then sending them to these laboratories has been used as a solution.

Currently, there are limited studies on effective sample storage solutions and optimal transportation time frame for patients with superficial fungal skin and nail infections. Therefore, this study aimed to develop equipment in the form of sample storage envelopes and also aimed to evaluate the efficacy of these envelopes in preserving skin and nail samples for subsequent microscopic examination and culture in cases with superficial fungal infections. Furthermore, this study sought to assess the appropriate time frame for sample transportation using this equipment.

## 2. Materials and Methods

The study included adult patients 18 years or older who presented at the Dermatology Clinic, Siriraj Hospital, between June 2021 and June 2022. Eligible patients were those clinically suspected of having superficial fungal infections of the skin or nails. Patients with coexisting pathogens (e.g., parasites and demodex) identified on the KOH examination, and patients who had received topical or oral antifungal agents within the preceding 1 month were excluded. This study was approved by the Siriraj Institutional Review Board of the Faculty of Medicine of Siriraj Hospital, Mahidol University (ECOA: SI 444/2021). Informed consent was obtained from all participants.

### 2.1. Sample Collections

The samples were collected from patients who were clinically suspected of having superficial fungal infections of the skin or nails. Based on fungal culture results, dermatophyte predominates in majority of patients diagnosed with superficial fungal infection of skin or nails, resulting in a high ratio of dermatophyte in the samples compared to nondermatophytes. Sample collection from skin lesions involved the following steps: First, the lesions were cleaned using gauze soaked with 70% alcohol to remove coexisting bacteria. The skin scraping was then performed using a surgical blade to collect the samples equally; one part was placed on a glass slide for immediate 20% KOH examination and fungal culture on Sabouraud dextrose agar (SDA) with chloramphenicol and SDA with chloramphenicol and cycloheximide at 26°C as a reference baseline, and the other four parts were placed in separate sample storage envelopes. Subsequent 20% KOH examination and fungal culture were performed from the stored samples in the envelopes on Day 3, Day 7, Day 14, and Day 28 using the same method.

**Figure 1:**
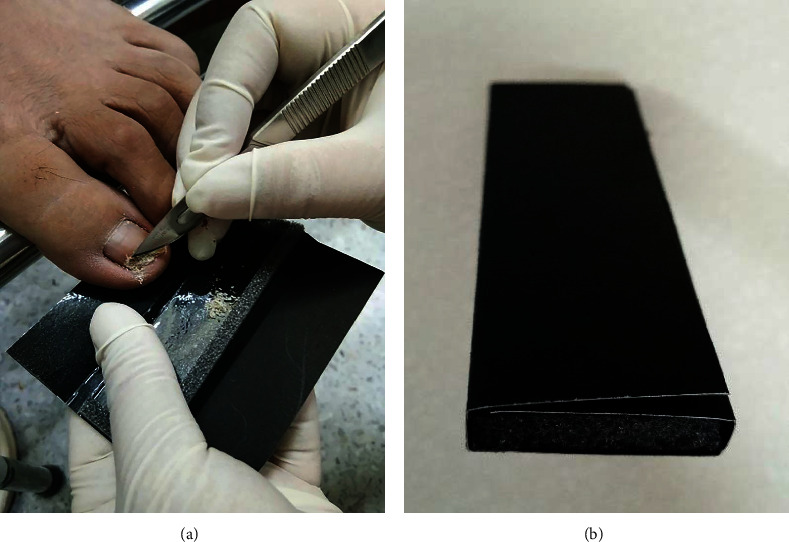
(a) Example of the sample collection process from a suspected nail sample into the sample storage envelope; (b) sample storage envelope after folding and closing.

For suspected fungal-infected nails, the nail plates and nail folds were cleaned with 70% alcohol to remove coexisting bacteria. Subsequently, the nails were cut to the junction between the lesions and the normal nail. The nail scraping was then performed using a surgical blade to collect the samples equally; one part was placed directly on a glass slide for an immediate 20% KOH examination and fungal culture. The other four parts were stored in separate sample storage envelopes (Figure 1(a)). Subsequent 20% KOH examination and fungal cultures were performed from the samples in envelopes on Day 3, Day 7, Day 14, and Day 28, following the same methods used for skin samples.

The envelopes were made of black cardboard, expanded polyethylene (EPE) foam, double-sided adhesive tape, and coated with transparent plastic to reduce adhesion between the envelope and the samples. Prior to sample collection, the envelopes were disinfected with 70% alcohol and left to dry for 30 min. The envelopes were then folded and closed (Figure 1(b)) and stored in zip-lock plastic bags at room temperature.

**Figure 2:**
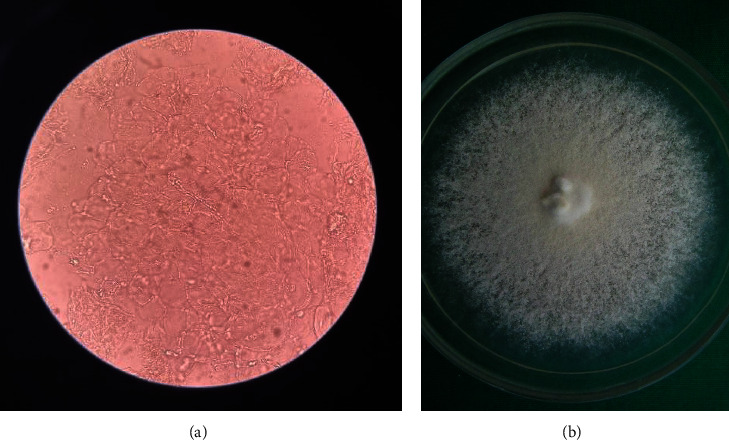
(a) KOH examination of branching septate hyphae at 40x magnification. (b) *Trichophyton mentagrophytes* growth at room temperature.

Fungal identification was based on the consensus agreement of three experienced fungal microscopists/technicians with more than 5 years of expertise in the mycological laboratory work. An example of KOH examination and fungal culture is shown in Figures 2(a) and 2(b), respectively.

### 2.2. Statistical Analysis

Descriptive statistics were used to describe demographic data, location of the lesions, duration of the disease, KOH examination, and fungal culture results on Day 0. Normally distributed continuous data were presented as mean ± standard deviation (SD), non-normally distributed continuous data as median and interquartile range (IQR), and categorical data as number and percentage.

Diagnostic accuracy indices including accuracy, sensitivity, specificity, positive predictive value (PPV), and negative predictive value (NPV) of KOH examination and fungal culture were assessed at different time intervals. The result at baseline (Day 0) was used as the standard reference point for these calculations. The results were reported as percentages along with 95% confidence interval (CI).

All statistical analyses were performed using PASW Statistics for Windows, version 28.0 (SPSS Inc, Chicago, Illinois, United States of America).

## 3. Results

A total of 90 patients were enrolled in the study, with 45 patients each suspected of having superficial fungal infections of the skin and nails.

### 3.1. Superficial Fungal Skin Infection

Among 45 cases suspected of superficial fungal skin infections, the mean age ± SD was 54.2 ± 16.5, and 19 (42.2%) patients were men. A total of 17 (37.8%) patients reported having pets. The distribution of the locations of the lesions was as follows: feet in 18 (40%) patients, trunk in 18 (40%) patients, groin/buttock in eight (17.8%) patients, and face in one (2.2%) patient. The median duration of the infection (IQR) was 5 (1, 12) months.

The KOH examination on Day 0 showed positive branching septate hyphae in 36 (80%) patients. Across all different time intervals, the accuracy, sensitivity, specificity, PPV, and NPV of the KOH examination were found to be 100%, as shown in Table 1.

The results of the fungal culture on Day 0 revealed dermatophyte growth in 27 (60%) patients, nondermatophyte molds in seven (15.6%) patients, and no growth or contamination in 11 (24.4%) patients. Among the dermatophyte group, *Trichophyton rubrum* was identified in 11 (40.7%) patients, *Trichophyton mentagrophytes* in nine (33.3%) patients, *Microsporum canis* in six (22.2%) patients, and *Trichophyton violaceum* in one (3.7%) patient. In the nondermatophyte mold group, *Neoscytalidium dimidiatum* was identified in six (85.7%) patients, while *Aspergillus niger* was identified in one (14.3%) patient.

**Figure 3:**
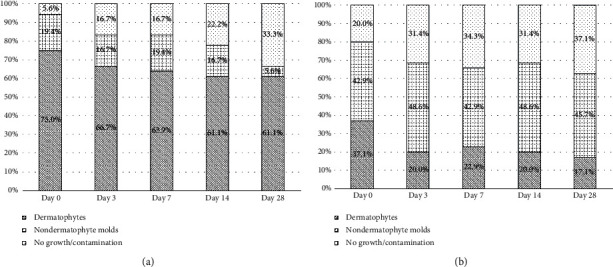
(a) Proportion of fungal culture results at different time intervals in superficial fungal infection of the skin cases with positive KOH examination on Day 0. (b) Proportion of fungal culture results at different time intervals in superficial fungal infection of the nail cases with positive KOH examination on Day 0.

The proportion of fungal culture results at different time intervals in suspected fungal skin infection cases with positive KOH examination on Day 0 is shown in Figure 3(a). As time progresses, the percentage of both dermatophytes and nondermatophytes decreases from 75.0% to 61.1% and 19.4% to 5.6%, respectively, with an increasing rate of no growth/contamination from 5.6% to 33.3%.

**Figure 4:**
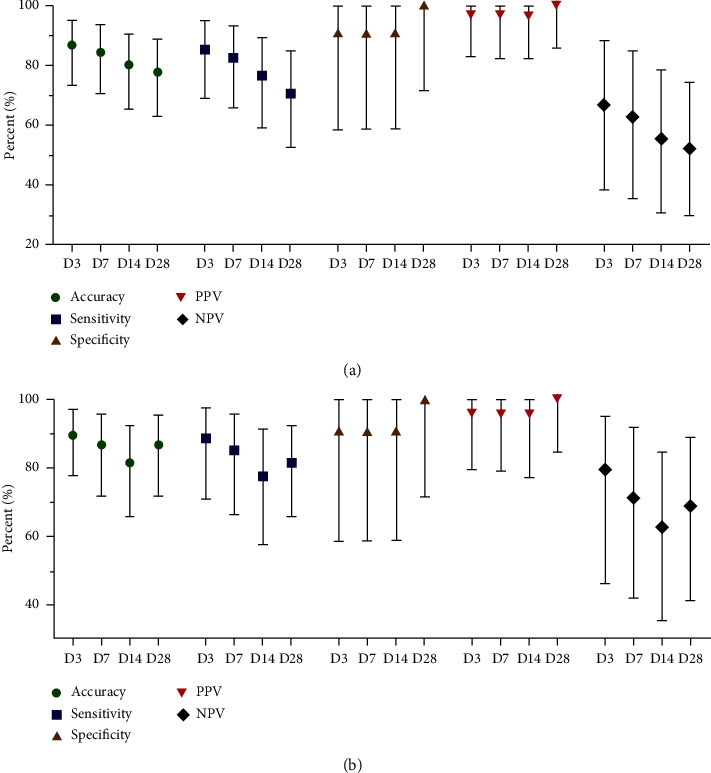
(a) Diagnostic accuracy of fungal culture from skin samples at different time intervals compared with fungal culture at Day 0. (b) Diagnostic accuracy of fungal culture from skin samples at different time intervals compared with fungal culture at Day 0, in the subgroup of cases with positive growth of dermatophytes at Day 0.

The results of the fungal culture analysis in different time intervals compared to the fungal culture on Day 0 are illustrated in Figure 4(a). Accuracy, sensitivity, and NPV tended to decrease over time. However, specificity and PPV remained high across all intervals, ranging from 90.9% to 100.0% and 96.3% to 100.0%, respectively. Further analysis of the subgroup of cases with positive growth of dermatophytes on Day 0 showed a similar trend, with higher accuracy, sensitivity, and NPV, as depicted in Figure 4(b).

### 3.2. Superficial Fungal Nail Infection

In the fungal nail infection group, the mean age ± SD was 60.8 ± 17.9, and 19 (42.2%) patients were male. Fifteen (33.3%) patients reported having pets. The samples were collected from toenails in 34 (75.6%) patients, of which 33 of 34 (97.1%) were from big toenails. The remaining 11 (24.4%) samples were collected from the fingernails, with the fourth fingernail being the site most commonly sampled (45.5%). The median duration (IQR) of the diseases was 12 (6, 24) months.

The KOH examination on Day 0 showed positive branching septate hyphae in 35 (77.8%) patients. The diagnostic accuracy of the KOH examination in suspected fungal nail infection cases is presented in Table 1. Similarly to the results in the fungal skin infection group, in all different time intervals, the accuracy, sensitivity, specificity, PPV, and NPV of the KOH examination compared to the KOH examination on Day 0 were found to be 100%.

Fungal culture results at Day 0 revealed the growth of dermatophytes in 13 (28.9%) patients, nondermatophyte molds in 15 (33.3%) patients, and no growth or contamination in 17 (37.8%) patients. Among the dermatophyte group, *T. rubrum* was identified in eight (61.5%) patients, *T. mentagrophytes* in four (30.8%) patients, and *T. tonsurans* in one (7.7%) patient. In the nondermatophyte mold group, *Aspergillus* spp. was identified in 11 (73.3%) patients, while *N. dimidiatum* and *Fusarium* spp. were identified in two (13.3%) patients each.

The proportion of fungal culture results at different time intervals in cases with positive KOH examination of nail samples on Day 0 is shown in Figure 3(b). As time progresses, the percentage of dermatophytes detection decreases from 37.1% to 17.1%, while the percentages of nondermatophyte molds remain relatively stable, ranging from 42.90% to 48.60%, and the percentages of no growth/contamination increase from 20.0% to 37.1%.

**Figure 5:**
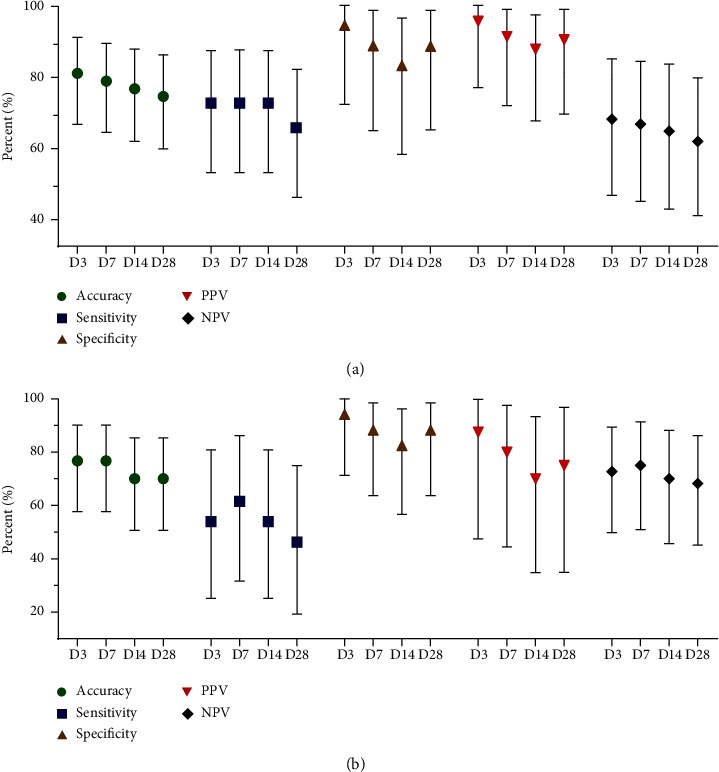
(a) Diagnostic accuracy of fungal culture from nail samples at different time intervals compared with fungal culture at Day 0. (b) Diagnostic accuracy of fungal culture from nail samples at different time intervals compared with fungal culture at Day 0, in the subgroup of cases with positive growth of dermatophytes at Day 0.

The analysis of fungal culture at different time intervals compared to fungal culture on Day 0 is illustrated in Figure 5(a). Accuracy, sensitivity, specificity, PPV, and NPV showed a tendency to decrease with time. However, specificity and PPV still remained high, at 88.2% and 90.0%, respectively, on Day 28. Further analysis of the subgroup of cases with positive growth of dermatophytes on Day 0 revealed a similar trend, with lower accuracy, sensitivity, specificity, PPV, and NPV, as shown in Figure 5(b).

## 4. Discussion

Direct microscopic examination and fungal culture are routinely performed in diagnostic investigations for superficial fungal skin and nail infection [6–8]. Investigations are recommended to confirm the diagnosis of this infection before starting treatment [8]. However, in many countries, access to fungal laboratories, particularly those capable of conducting fungal culture, is limited to only some tertiary hospitals [5, 9–11]. This study presented sample storage envelopes for sample transportation. Moreover, this study demonstrated a diagnostic accuracy of 100% for up to 28 days with KOH examination. Regarding fungal culture, the results showed a high specificity that exceeded 0.80 on Day 28 for both skin and nail samples. This suggests that prompt sample transfer within 28 days is not only feasible but also ensures reliable results.

In this study, the samples were stored at room temperature due to facilitation for relocation. In addition, in clinical practice, sample collection and storage were conducted at room temperature. According to the previous study, all specimens from skin scrapings were stored using the adhesive tape technique at room temperature, the PPV of fungal cultures at Day 3 or 4, 7, 14, and 28 compared to fungal culture at Day 0 were 96.9%, 95.3%, 96.9%, and 97.4%, respectively. The positive predict value of fungal culture at different intervals remained very high over the period of time [6].

In regions with limited qualified laboratories, some hospitals need to collect samples and transfer them to tertiary hospitals to perform the KOH examination and fungal culture. This study addresses the challenges associated with delayed fungal microscopy and culture in newly obtained samples. In response, this study presented sample storage envelopes made from black cardboard, EPE foam, double-sided adhesive tape, and a transparent plastic coating, which proved to be an efficient method of preserving the integrity of skin and nail samples for subsequent diagnostic evaluations. In addition, the envelope design was used to prevent samples from becoming dislodged during the collection process. The use of black materials also aids in the easy identification of scales collected by scraping. To our knowledge, previous studies have explored swab transport systems to maintain the viability of *Candida* spp. and other pathogenic fungi, which showed effective results [12, 13]. However, these studies were conducted in vitro and focused on viability for up to only 48 h. In addition, such swab transport systems may not be suitable for superficial fungal infections of the skin and nails, which require sample collection using techniques such as scraping with a surgical blade or tape stripping [14].

The sources of contamination can be from environmental contaminants such as nonpathogenic fungi from air, surfaces, or materials during collection. The proportion of fungal culture results at different time intervals in suspected fungal skin infection cases with positive KOH examination on Day 0 is shown in Figure 3. According to the results of the study, the percentage of fungal culture results in contamination/no growth in suspected fungal skin infection of the skin and nail cases was 5.6% and 20.0% at Day 0 with an increasing rate of 33.3% and 37.1% at Day 28, respectively.

Improper collection techniques such as inadequate sanitization, improper handling of instruments, or failure to use sterile containers can all lead to contamination. Accordingly, in this study, we only performed specimen collection by expert microbiologists who expertise in collecting specimen from the skin and nails to avoid contamination. Prevention strategies included sterilization before collecting samples, using sterile equipment, proper technique, and collecting specimen in the laboratory setting to avoid environmental contaminants.

Although there is a patent for a fungal skin infection diagnostic kit box designed to transfer samples to remote locations [15], and there are commercially available inventions specifically designed to collect and transport skin and nail samples that have also been recommended for use in the literature [16], the clinical efficacy of their uses over different time intervals has not been addressed. The advantages of the current study lie in the clinical validation of our invention's efficacy across different time intervals, which provide evidence of the practical benefits of our sample storage envelopes in a real-world clinical setting. In addition, our sample storage envelope design is made from readily available and inexpensive materials, and the design allows the envelope to be easily cleaned.

The results of this study demonstrate that the KOH examination of the samples stored using our envelopes achieved diagnostic accuracy, including accuracy, sensitivity, specificity, PPV, and NPV of 100% for up to almost 1 month compared to the KOH examination on Day 0, regardless of whether the samples were from the skin or nails. When comparing our findings with a previous study using the adhesive tape technique for sample collection and subsequent KOH examination of skin samples [6], similar results were observed but with a slightly higher diagnostic accuracy.

In terms of fungal culture results, this study observed a declining trend in diagnostic accuracy as time progressed for both skin and nail samples. However, the specificity and PPV of the test for skin samples remained consistently high, even up to Day 28, both at 100%, which aligns with the findings of a previous study that reported values of 97.7% and 99.1%, respectively [6]. Although the sensitivity of fungal culture and NPV decreased with time, this study provided a higher sensitivity and NPV at later time points. In the current study, sensitivity and NPV were 82.35% and 62.50% on Day 7, and 70.59% and 52.40% on Day 28, while the previous study reported values of 68.6% and 51.9% on Day 7, and 31.4% and 34.7% on Day 28, respectively [6]. The declining trend in sensitivity and NPV of fungal cultures could be attributed to the lack of keratin and a dry environment in the stored samples, which could lead to fungal death and subsequently affect the sensitivity of the fungal culture. However, the higher sensitivity in the current study compared to the previous study could be attributed to the possible deprivation of oxygen due to the use of adhesive tapes, which might lead to increased fungal death in the previous study. On the contrary, KOH examination can detect dead fungi, contributing to its higher sensitivity in later time intervals.

Furthermore, subgroup analyses of skin and nail samples, focused solely on dermatophyte infection at baseline, were performed to assess the diagnostic accuracy of the fungal culture at different time intervals. The results indicated an overall decreasing trend in diagnostic accuracy for both skin and nail samples over time, similar to the findings from the overall analysis. However, in the analysis of the nail sample group, the diagnostic accuracy, especially the sensitivity, was notably lower compared to the analysis of skin samples. This difference may possibly be due to the predominance of nondermatophyte molds or contaminations in nail samples, which may lead to an increased rate of false-negative results.

There were several limitations in this study. First, this study only examined the results of using our sample storage envelopes for a limited period of up to 28 days. The data indicated a decrease in the diagnostic accuracy of fungal cultures over time, suggesting that this simple storage method may not be suitable for long-term storage of samples. Our results emphasize the need for prompt sample transfer. However, the study was conducted within the typical 28-day time frame for sample transfer. More studies with longer study periods should be conducted to address these issues. Second, more advanced diagnostic methods, such as PCR for skin, hair, or nail samples, or histopathological examination of periodic acid-Schiff (PAS)–stained nail clippings, have gained popularity. However, this study did not investigate the efficacy of sample storage envelopes on these methods. More studies are required to evaluate the efficacy of envelopes for these advanced diagnostic methods.

## 5. Conclusion

In conclusion, this study proposes a practical and simple method of storing samples in cases of superficial fungal infections. The sample storage envelopes effectively preserve skin and nail samples, maintaining diagnostic accuracy for up to 28 days with KOH examination. However, for fungal culture, diagnostic accuracy decreases over time, emphasizing the need for prompt transfer of samples when culture results are required. However, given the consistently high specificity exceeding 0.80 on Day 28 for skin and nail samples, transferring samples within 28 days remains a reliable practice.

## Figures and Tables

**Table 1:** Diagnostic accuracy of KOH examination at different time intervals in cases with suspected superficial fungal infections of the skin and nails.

	Accuracy (%) (95% CI)	Sensitivity (%) (95% CI)	Specificity (%) (95% CI)	PPV (%) (95% CI)	NPV (%) (95% CI)
*Cases with suspected superficial fungal infections of the skin (N = 45)*					
Day 3, 7, 14, and 28	**100.0** (92.1–100.0)	**100.0** (90.3–100.0)	**100.0** (66.4–100.0)	**100.0** (90.3–100.0)	**100.0** (66.4–100.0)

*Cases with suspected superficial fungal infections of the nails (N = 45)*					
Day 3, 7, 14, and 28	**100.0** (92.1–100.0)	**100.0** (90.0–100.0)	**100.0** (69.2–100.0)	**100.0** (90.0–100.0)	**100.0** (69.2–100.0)

*Note:* The bold values do not represent a *T*-value. Either the bold or unbold version of the values is acceptable.

## Data Availability

The data that support the findings of this study are available from the corresponding author upon reasonable request.
